# Some Noticeable Features

**Published:** 1920-10-16

**Authors:** 


### THE LIVERPOOL TUBERCULOSIS CONFERENCE.

### Some Noticeable Features.

The medical conference is by now a familiar feature of public life. It has even got into fiction, as in a French novel where a pushing young *entrepreneur* is represented as advertising himself by pretending to be in organising charge of one. And just as most people of middle age have their well-marked little characteristics, so there are certain features always noticeable about medical conferences. To begin with, no matter what the special subject, the most prominent in the local medical world must be asked to say their say thereon. So it was at Liverpool; and one said that the only way the tubercle bacillus could enter a child's body was in milk. Some of those present must have longed for a sight of the astonishment of Dr. Clive Rivière and other workers on hilus tubercle, at hearing such a dictum. Then there are many hardy annuals which must come on exhibit, if only because they are such precious items of public propaganda that no chance to display them may be omitted. These we also had, from other lips likewise left anonymous. And the distinctive things about this congress may be put thus, that there was one likely suggestion and one serious omission.

The suggestion was Professor Beattie's. He spoke of a method, not yet fully tested, of killing *B.* tuberculosis in milk, without the use of high temperature, and in bulk, by means of electricity. One must discount this by the fact that even in juvenile tubercle, and even (a statement to the contrary notwithstanding) in non-pulmonary juvenile tubercle, the bovine variety of the germ is less common than the human one. Also practical difficulties will occur to the layman. Many industrial districts are studded with small dairy farms not much bigger than small holdings. But for big towns, and particularly with that desideratum municipal milk supplies, a good means of bulk sterilisation might mean something substantial in the way of prophylaxis.

Nevertheless, the omission was a vastly bigger affair. Beyond a speech by a Glasgow civic dignitary, and an allusion by Professor Benjamin Moore, there was scant mention of the supreme practical importance of the sociological aspect of this disease. In face of the fact that tuberculosis is a disease of "social misery" (if that facile rendering of the expressive French *misère* may be allowed), that it is four times commoner in the working class than in the middle class, it is a mere evasion, seeing the patent want of success of the current anti-tuberculosis campaign, going on lecturing laymen about gross infection or even carelessness of hygiene—especially when they retaliate by going to Harley Street about five in the morning and looking almost in vain for an open bedroom window, at all events for a well-opened one. The obvious practical and foremost indication for action is social amelioration. Give the working class middle class conditions of life, and in proportion to your success in that endeavour will be your anti-tuberculosis victory. It is in vain to say that the existing measures of prophylaxis are not carried out. They are not carried out because of economic difficulties which are insurmountable under the economic conditions of this century. Even if they were carried out, it may well be doubted whether one could be sure of their efficiency. Long ago Professor Karl Pearson showed that tuberculosis in Aberdeen, at a period when that city was without tuberculosis dispensaries, had declined more quickly than in Edinburgh, the British headquarters of the dispensary movements. Not that we would decry these institutions in the slightest: they alone afford the clinical material which can ripen a man into a true tuberculosis specialist, whose clinical activity is of great value. But with regard to broad prophylaxis, matters are as has been endeavoured to set out above. This is the lesson of the war, as others besides ourselves have often urged, and what is more important still, this is the lesson too of unbiased reason. (See also p. 57.)

